# The Attitudes and Intention to Participate in Hemoglobinopathy Carrier Screening in The Netherlands among Individuals from Turkish, Moroccan, and Surinamese Descent

**DOI:** 10.1155/2013/374831

**Published:** 2013-11-17

**Authors:** Sylvia M. van der Pal, Nicole M. C. van Kesteren, Jacobus P. van Wouwe, Paula van Dommelen, Symone B. Detmar

**Affiliations:** ^1^Department of Child Health, Netherlands Organization for Applied Scientific Research (TNO), P.O. Box 2215, 2301 CE Leiden, The Netherlands; ^2^Department of Life Style, Netherlands Organization for Applied Scientific Research (TNO), P.O. Box 2215, 2301 CE Leiden, The Netherlands

## Abstract

*Objective*. To explore factors that influence intention to participate in hemoglobinopathy (HbP) carrier screening under Dutch subjects at risk, since HbP became more common in The Netherlands. *Method*. Structured interviews with 301 subjects from Turkish, Moroccan, or Surinamese ethnicity. *Results*. Half of the participants were familiar with HbP, 27% with carrier screening. Only 55% correctly answered basic knowledge items. After balanced information, 83% percent of subjects express intention to participate in HbP carrier screening. Intention to participate was correlated with (1) anticipated negative feelings, (2) valuing a physician's advice, and (3) beliefs on significance of carrier screening. Risk perception was a significant determinant, while respondents were unaware of HbP as endemic in their country of birth. Respondents preferred screening before pregnancy and at cost < 50€. *Conclusion*. These findings show the importance of informing those at risk by tailored health education. We propose easy access at no costs for those willing to participate in HbP carrier screening.

## 1. Introduction

Hemoglobinopathy (HbP) is a genetic mild to acute anemia. The two common forms, thalassemia and sickle cell anemia, occur in Africa, the Mediterranean Basin, and Southeast Asia. Carriers of HbP have an evolutionary benefit in these areas where malaria is endemic. 

A simple blood test reveals that if both partners are carriers, they have a 25% chance in each pregnancy for a newborn with HbP. If (future) parents are at risk, they can prevent the birth of a HbP baby, or they can decide not to have more children, or consider prenatal or preimplantation diagnosis. 

Strategies to offer HbP carrier screening differ worldwide. Some Middle Eastern countries have nationwide premarital carrier screening programs [1–8]. In Northern Cyprus, with a high prevalence of thalassemia, premarital carrier screening is mandatory since 1980 [9]. This screening is considered effective in terms of the reduction of children affected with HbP; after 1984 the number of newborns with thalassemia in Northern Cyprus dropped from 18–20 to 6-7 annually. Since 1991 only 5 babies with thalassemia were born [9]. 

Prevalence of HbP has increased in Northern Europe because of migration [10]. This raises questions as to whether it is beneficial to offer carrier screening and whether ethnically targeted screening is also an option in The Netherlands [11]. HbP carrier screening is not routinely offered to high risk groups even though there are sufficient options to offer neonatal, prenatal, or even preconception carrier screening in The Netherlands [12]. A recent Dutch study showed feasibility and desirability of a combined preconception ancestry-based carrier screening for cystic fibrosis (CF) and HbP [13, 14]. 

Since 2007 all Dutch newborns are screened for sickle cell anemia in the neonatal screening program. The method used also reveals carriers. Parents have to choose to receive the result. In the first year of the extended screening, 64 newborns were identified with sickle cell anemia (prevalence 0,035%) [15]. In addition, 806 carriers of sickle cell anemia (prevalence 0,4%) were found. Only few parents did show up for genetic counseling in the regional clinical genetic centers at Amsterdam and Rotterdam, the cities with the largest migrant populations in The Netherlands [16]. The information on neonatal carrier for sickle cell anemia and the implications for (future) parents was suboptimal and parents also are occasionally ill-informed [17, 18]. Furthermore, the disclosure of a newborn carrier may lead to confusion, guilt, anxiety, and early stigmatization and it may even reveal nonpaternity [19]. Ethical issues are at stake regarding timing and extent of disclosure towards the affected child and other family members [20]. 

The experience with Dutch HbP carrier screening shows the importance of correctly informing high risk individuals and taking into account their familiarity with HbP. This study with data of 2007 aims to explore (a) the familiarity with HbP and HbP carrier screening among high risk groups in The Netherlands; (b) the social-psychological determinants of the intention of these high risk groups to participate in HbP carrier screening; and (c) their preferences regarding HbP carrier screening. 

## 2. Materials and Methods

### 2.1. Subjects

Structured interviews by a standard questionnaire were performed in 301 Dutch subjects living in or around Rotterdam of Turkish (*n* = 100), Moroccan (*n* = 100), or Surinamese (*n* = 101) ethnicity (see Table 1 for demographics). These interviews lasted 29–156 minutes (depending on the questions asked by the subjects) and were held at home or in a nearby community center by an interviewer of matching ethnicity in Dutch and their native language. All subjects aged 18–45 participating in a community network of a multicultural research center were invited. Respondents were asked for names to recruit new potential participants within Rotterdam. Subjects were selected to evenly represent males-females, various age intervals, and those living in different parts of town (SES equivalent). New applicants were entered until we reached three groups of 100 participants. All participants received a gift certificate after the interview of 20€.

### 2.2. Interview

**Figure 1:**
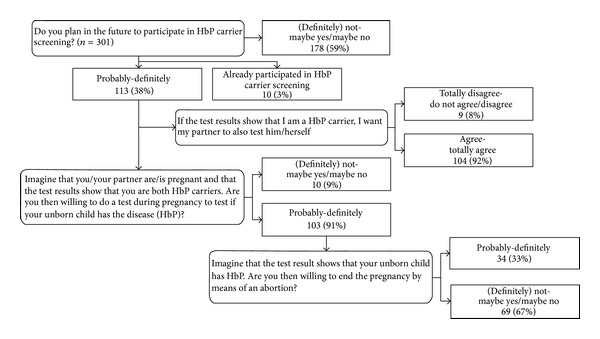
Flow chart intention to participate in HbP carrier screening.

The interviewers followed a constructed questionnaire with the following issues:

- *knowledge* on HbP (true or, resp., false/yes or no), carrier screening, and perceived carrier risk (5 scales of (dis)agreement), based on the Precaution Adoption Process Model [21];
- a short explanation of HbP and its hereditary nature;
- reaction to various HbP carrier screening situations, by asking *intentions*  and hypothesized actions in explicit scenarios (see Figure 1);
  1. “Imagine you participated in carrier screening and the test shows you are a carrier”;
  2. “Imagine you participated in carrier screening since you're pregnant and results show you're both carriers”;
  3. “Imagine your unborn child is diagnosed with HbP and you're both carriers”;
- items on determinants of carrier screening *behavior*, based on the Theory of Planned Behavior (TPB) [22] and Health Belief Model (HBM) [23]. These assess risk perception, attitudinal beliefs, social norms, self-efficacy, and intentions. Positive and negative anti-cipated affective reactions to HbP carrier screening which may influence the respondents' intention to participate in carrier screening (see Table 2), as shown in other studies on screening behavior [24, 25] (all answers with 5 scales of (dis)agreement);
- *preferred screening conditions:* respondents were asked eight times during the interview to select one of the two hypothetical screening situations (two vignette cards each representing five specific conditions requiring HbP carrier screening; choose appropriate card). The conjoint analysis method forces respondents to make tradeoffs between different attributes of hypothetical situations. We distinguished five attribute categories and varied these for each vignette card:
  1. timing: while in high school/with pregnancy wish/at beginning of pregnancy;
  2. initiative or approach: by invitation/self-made decision;
  3. health education and counseling about the test result: by group/individual, in Dutch/in other preferred languages;
  4. who will test and communicate its result: midwife/health care office/general physician;
  5. financial contribution: 0/50/100/200 euro;
- *other aspects* such as religious belief, family structure and cultural identity (footnote of Table 2), preference if carrier screening would be offered more commonly, and open-end questions on the respondent's intention to participate in HbP carrier screening. The background variables obtained were determinants of the model (Table 1).

### 2.3. Analyses

Data was analyzed using SPSS Version 14.0 and R Version 2.7.2. First, the demographics of the respondents were compared with the demographics of the Dutch population matched by ethnicity. Descriptive analyses were performed to explore the percentage of respondents per answer category. Within scenario 1 (“Imagine you participated in HbP carrier screening and the test shows you are a carrier”) multivariate backwards regression analysis was performed to explore determinants correlating with the *intention to participate* in testing. Finally, for conjoint analysis of the two vignette cards, multilevel logistic regression analysis (random intercept) was performed with the *preferred* hypothetical *screening circumstances* as outcome variable and a combination of dummy variables as independent variables. As an example we show the equation of the model with three attributes (*A*, *B*, and *C*) each with three levels (1, 2, or 3). *A*1 is attribute *A* with level 1. *I*_*A*1_ is a dummy variable, which is 1 if attribute *A* has level 1 or 0 if not. For instance, situation 1 (*s*1) = {*A*1, *B*2, *C*2} and then *I*_*A*1_(*s*1) = 1, *I*_*A*2_(*s*1) = 0, and *I*_*A*3_(*s*1) = 0. We assume that the value (*V*) of *s*1 is a linear function of the values of the attributes: (*s*1) = *β*_0_ + *β*_11_∗*I*_*A*1_(*s*1) + *β*_12_∗*I*_*A*2_(*s*1) + (−(*β*_11_ + *β*_12_))∗*I*_*A*3_(*s*1) + *β*_21_∗*I*_*B*1_(*s*1) + *β*_22_∗*I*_*B*2_(*s*1) + (−(*β*_21_ + *β*_22_))∗*I*_*B*3_(*s*1) + *β*_31_∗*I*_*C*1_(*s*1) + *β*_32_∗*I*_*C*2_(*s*1) + (−(*β*_31_ + *β*_32_))∗*I*_*C*3_(*s*1). The equation of the model is *V*(*s*2) − *V*(*s*1). The respondent has to choose which situation (one or two) he/she prefers (*V*(*s*2) − *V*(*s*1) > 0 or *V*(*s*2) − *V*(*s*1) < 0). The beta's are estimated in the model. A multilevel model was used because each respondent made this choice eight times. We used a reference level that resembles foreign screening programs and is suitable to be introduced within the Dutch health system.

## 3. Results 

### 3.1. Subjects

Demographics of the 301 participants (Table 1) show that they are less often married and more often highly educated, compared to the Dutch population of matched ethnicity (Statistics Netherlands CBS, http://www.cbs.nl/).

### 3.2. Knowledge

Half of the participants (45%) were familiar with HbP (sickle cell disease or thalassemia), partly because they knew someone with HbP (39%, *n* = 53 of 135). Those from Surinamese ethnicity more frequently were aware of HbP (61%) and knew somebody with the condition (47%, *n* = 29 of 62). A much smaller group (27%) was familiar with carrier screening. Of this group, ten already participated in HbP carrier screening (1 a carrier, 1 forgot the test result), seven considered carrier screening, and 64 were not interested in screening.

Half of the participants (55%) correctly answered that even if both parents are carriers a healthy baby is possible, 44% identified the statement “carriers get sick” to be incorrect, 43% identified the statement “mainly women transfer genetic diseases to their children” to be incorrect, and only 25% correctly identified the statement “HbP is more common in my country of birth.” Those of Surinamese background scored higher on knowledge (5.84 of a scale 0–10) compared to ethnic Turkish or Moroccan participants (4.76, resp., 3.78).

### 3.3. Behavior

More than one-third (38%) of the participants expressed intention to participate in carrier screening, 3% were tested, and 59% were in doubt or disregarded testing. The intention to participate was higher among females (46.6% versus 30.5%). A flow chart displays the responses by the three different scenarios (Figure 1). While 103 respondents intend to participate in prenatal screening, only 34 of them opt for abortion if HbP is diagnosed. The average negative attitude towards abortion is scored 1.97 (on a scale from 1, negative, to 5, positive). After the first trimester of pregnancy this score is even 1.52.

### 3.4. Intention to Participate

All determinants for intention to perform a HbP carrier test (footnote Table 2) were added in the multiple regression, and the question “Will you plan to participate in future HbP carrier screening?” was a dependent variable. In the model four significant (*P* < 0.01) variables were obtained (*R* ^2^ 0.27). These were (1) beliefs about (the importance of) HbP carrier screening (*P* < 0.001), (2) risk perception to be a carrier or to have a child with HbP (*P* = 0.01), (3) anticipated negative feelings (*P* = 0.02), and (4) it is relevant for your health to listen to a physician and follow his advice (*P* = 0.04).

### 3.5. Preferred Screening Circumstances

Conjoint analysis showed that the respondents preferred screening before pregnancy instead of during pregnancy (screening during pregnancy versus screening before; odds ratio = 0.78, 95%, confidence interval = 0.71–0.87). Those with Turkish ethnicity preferred screening earlier, at school age. Screening free of charge or for less than 50 euros is preferred. The current screening test in The Netherlands costs 50€ and a full diagnostic test 100–500€. These costs are not provided by standard health insurance.

### 3.6. Other Aspects

Respondents disagreed with the statement “I dislike HbP carrier screening by reference to ethnicity” (mean score of 2.16 on a scale from 1, totally disagree, to 5, totally agree). They agreed with the statement “I am more intended to get screened if I know HbP to be common in my country of birth” (mean score 4.14). 

The open-end questions finally revealed that important motivations to participate in carrier screening are “perceived relevance for future children” and “importance of health issues;” while reasons not to participate are “feeling healthy, no pregnancy wish, and no family member with HbP.” Ninety-two percent of the respondents would inform their family (“they have a right to know”) and 98% would inform their partner (“right to know, to get tested too, and matter of honesty”). Seventy-eight percent of the respondents agree that HbP carrier screening may be offered routinely. Most respondents prefer information on a leaflet sent by mail in the Dutch language or to be informed by general practitioners. They appreciated being informed by the interview, and some stated: “it is interesting, I learned something.” 

## 4. Discussion

Less than half of those from Surinamese, Moroccan, and Turkish ethnicity in The Netherlands are familiar with HbP (45%) and fewer with carrier screening (27%). Half expressed intention to be tested (41%). Those from Surinam were relatively more familiar and knew someone with sickle cell anemia. Only 25% of subjects knew HbP to be endemic in their country of birth. 

A recent study found that 56% of Dutch recently married couples had a positive intention to participate in CF carrier screening [26]. Van Elderen et al. [27] showed a higher intention (83.5%) to participate in HbP carrier screening when planning for pregnancy, among 109 ethnic Turkish females in The Netherlands. We found lower intention to participate in those aged 18–45 (38%), although intention was higher among the women (46.6%). Our subjects could also score “maybe yes/maybe not”; 29.3% of the women choose this answer category. Eventually 26% of the women had no intention to participate in carrier screening. Van Elderen et al. [27] found that one-third (30.3%) of the women intended to opt for prenatal screening after carrier screening and we found a similar score (33%). 

### 4.1. Determinants of the Intention to Participate

We also examined social-psychological factors that influence the intention to participate in carrier screening. Respondents who had a more positive intention towards participating acknowledged the risk of being a carrier and were more inclined to listen to a physician's advice. Anticipated negative feelings with regard to carrier screening appeared to be another relevant factor. In other words, the more negative feelings are anticipated, the less one intends to participate in carrier screening. This result is consistent with previous findings on screening behavior, indicating that both cognitive and affective processes play a role in the decision to participate [25, 28].

### 4.2. Preferred Screening Circumstance

Our subjects show a clear preference for HbP carrier screening before pregnancy, profoundly shown in their choices with the situational cards (vignettes). Screening before pregnancy is chosen over screening during pregnancy. Those of Turkish ethnicity preferred screening even earlier: during school age as is customary in, for example, the Jewish communities in Israel, NY, and Australia [29]. While 91% of the respondents with an intention to participate opt for screening during pregnancy if both partners would be carriers, only a third of them would be willing to consider termination of pregnancy if the prenatal test results in a HbP baby. This finding is consistent with the UK, where one-third of identified “at high risk couples” chose to have prenatal diagnostic tests [30]. We did not ask why they would consider participating in prenatal screening if they are not willing to consider termination of pregnancy. However, the responses at the end of the interview showed that they would like to know their unborn child's status, mainly to be prepared for a sick child after birth. A recent qualitative study by Gitsels-van der Wal et al. [31] among pregnant Muslim women showed that beliefs associated with their religion played a significant role in decision-making regarding antenatal screening, particularly regarding termination of pregnancy. In our study we focused on the intention for prenatal screening during the hypothetic scenarios. There are other possibilities besides pregnancy termination, such as not having children, adoption, changing partners, and premarital screening. The results of this study show the importance of early prenatal testing to allow at risk couples to consider more options during the first trimester of pregnancy and importance to inform parents-to-be in the preconception phase.

### 4.3. Limitations and Strengths

This study measured intention to participate in HbP carrier screening in hypothetical scenarios, but “intention” may not match “actual behavior.” Also, most subjects had not previously heard about HbP and therefore had limited time to consider the pros and cons of such behavior. Because the snowball method was used to recruit participants, we obtained more highly educated respondents. Regression analyses in this study however showed that educational level was not significantly associated with the intention to participate in HbP carrier screening. Because of the matching ethnicity of respondent and interviewer, it was possible to directly answer and ask for further clarification in the native language. Finally, the sample used in this study warrants some concern, as our sample seems not entirely demographically representative for the larger population of Turkish, Moroccan, and Surinamese people in The Netherlands. Therefore, we need to be cautious in generalizing the results from this study to the total Turkish, Moroccan, and Surinamese population. 

### 4.4. Implications

For public health education these results imply the need for tailored health education to high risk groups (those of Surinamese, Moroccan, and Turkish ethnicity) on HbP carrier screening. Knowledge on HbP heritability, on the risk of being a carrier, on consequences, and on implications of a positive test result is of importance. Our subjects indicated that they would be more intended to screen if they knew that HbP is more common in their country of birth. Only 25% knew this to be the case indeed. It is therefore relevant to mention that HbP is endemic in the country of ethnicity in health education. Also the information that other diseases, such as CF, are more common in Caucasians is relevant. In health education the knowledge that most Middle Eastern countries introduced national screening programs to detect carriers and the fact that Islam fatwa allows abortion in the first trimester for medical reasons like HbP [32] are also important issues for health education. For those who are willing to participate easy access to screening should be facilitated. Recent research by Lakeman et al. [13, 14] showed that it is possible to offer a combination of HbP and CF carrier screening in The Netherlands, based on ethnicity. However, currently no population based carrier screening for HbP is offered in The Netherlands and most carriers are found due to unexplained mild anemia, during pregnancy of after neonatal screening. People who receive these carrier test results should be referred to clinical genetic centers that provide good quality information. 

## 5. Conclusion

These findings show the importance to inform those with high risk with tailored health education. For those willing to participate easy access to this type of screening should be facilitated. Because it is important for high risk groups that both health education and carrier screening will take place before pregnancy, health education should be focused during preconception. With a standard HbP carrier screening implemented, tailored health education, genetic counseling, and decision aids should receive attention. These should be developed in concordance with the level of knowledge and the preferences of those at high risk. 

## Figures and Tables

**Table 1:** Demographics of participants by ethnicity.

	Total *n* = 301	Turkish *n* = 100	Moroccan *n* = 100	Surinamese *n* = 101
Age, mean (SD) range: 18–45 years	33.4 (7.5)	32.7 (7.6)	33.4 (7.4)	34.2 (7.4)
Gender, *n* (%)				
Male	144 (48%)	50	45	49
Female	157 (52%)	50	55	52
Partner, *n* (%)				
Yes, married	126 (42%)	59	51	16
Yes, living together	23 (8%)	1	3	19
No	152 (50%)	40	46	66
Duration relationship in years, mean (SD)	11.5 (6.9)	12.1 (7.1)	10.9 (6.3)	11.7 (7.6)
Children, *n* (%)				
Yes	178 (59%)	65	53	60
No	123 (41%)	35	47	41
Country of birth, *n* (%)				
Turkey	70 (23%)	70	—	—
Morocco	84 (28%)	—	84	—
Surinam	57 (19%)	—	—	57
The Netherlands	90 (30%)	30	16	44
Country of birth father, *n* (%)				
Turkey	100 (33%)	99	1	—
Morocco	99 (33%)	—	99	—
Surinam	99 (33%)	—	—	99
The Netherlands	3 (1%)	1	—	2
Country of birth mother, *n* (%)				
Turkey	100 (33%)	100	—	—
Morocco	100 (33%)	—	100	—
Surinam	97 (32%)	—		97
The Netherlands	4 (1%)	—		4
Educational level (highest educational level obtained in The Netherlands; *n* = 213)				
Low	44 (21%)	15 (24%)	18 (28%)	11 (13%)
Intermediate	106 (50%)	26 (42%)	33 (51%)	47 (55%)
High	63 (30%)	21 (34%)	14 (22%)	28 (33%)
Educational level (highest educational level obtained in country of birth; *n* = 88)				
Low	22 (25%)	14 (37%)	7 (20%)	1 (7%)
Intermediate	52 (59%)	18 (47%)	22 (63%)	12 (80%)
High	14 (16%)	6 (16%)	6 (17%)	2 (13%)
Duration interview in minutes, mean (SD): range 29–156 min	60 (18)	63 (14)	59 (23)	58 (14)

**Table 2:** Determinants of the intention to participate in HbP carrier screening, univariate and multivariate unstandardized coefficients and 95% CIs.

		Univariate*¹*	*P* value	Multivariate^2^	*P* value
1	Attitudinal beliefs about screening	1.01 0.80–1.22	<0.001	0.95 0.75–1.16	<0.001
2	Anticipated positive feelings	0.23 0.11–0.36	<0.001		
3	Perceived importance of health	0.46 0.19–0.72	0.001		
4	Perceived social norm of screening	0.26 0.11–0.40	0.001		
5	Anticipated negative feelings	0.28 0.11–0.45	0.001	0.18 0.03–0.33	0.02
6	Gender (male = 1, female = 2)	0.35 0.08–0.62	0.01		
7	Perceived importance of physician's advice	0.15 0.03–0.28	0.02	0.12 0.01–0.23	0.04
8	Risk perception of being a carrier or having a child with HbP	0.27 0.004–0.28	0.02	0.25 0.06–0.43	0.01
9	Perceived importance of faith	0.14 0.05–0.48	0.04		

^1^Univariate correlation of intention with significant (*P* < 0.05) determinants, all determinants.

(1) Background information: age, gender, partner (married, living together, no partner), kids (yes, no), and educational level (finished with a certificate).

(2) Determinants measured with one or two items: profession or faith, perceived importance of faith, perceived importance of family (ties), perceived importance of adapting to the Dutch culture, perceived importance of maintaining own culture, perceived importance of health, perceived responsibility for own health, perceived importance of doctor's advice, perceived importance of fate/destiny, and perceived importance of a higher power/God which influences health.

(3) Multiple item scales within scenario 1 (“Imagine you participated in HbP carrier screening and it shows you are a carrier for HbP”): knowledge about HbP and carrier (screening) (5 items), anticipated positive feelings (2 items, Cronbach's alpha = 0.49), anticipated negative feelings (3 items, alpha = 0.52), attitudinal beliefs about HbP carrier screening (10 items, alpha = 0.73), perceived social norm of HbP carrier screening (7 items, alpha = 0.88), self-efficacy (trust in own abilities to participate in HbP carrier screening; 2 items, alpha = 0.65), and risk perception of being a carrier or having a child with HbP (4 items, alpha = 0.81).

^2^Backwards multiple regression analysis (Pin = 0.05 en Pout = 0.055) with the significant determinants of the univariate analysis. Adjusted *R* ^2^ = 0.27; only the significant determinants in the model are displayed.
